# Dacryoendoscopy as a frontier technology for lacrimal drainage disorders

**DOI:** 10.1007/s10384-025-01255-7

**Published:** 2025-08-07

**Authors:** Manabu Sugimoto, Yasushi Inoue, Atsushi Shiraishi

**Affiliations:** 1 Sugimoto Eye Clinic, 158-5 Makabe, Soja City, Okayama 719-1134 Japan; 2 Inoue Eye Clinic, Tamano, Okayama Japan; 3 Department of Ophthalmology, Ehime University Graduate School of Medicine, Toon, Ehime Japan

**Keywords:** Dacryoendoscope, Lacrimal passage reconstruction surgery, Nasolacrimal duct obstruction (NLDO), Congenital nasolacrimal duct obstruction, Canalicular obstruction

## Abstract

Dacryocystorhinostomy (DCR) for adult patients with acquired nasolacrimal duct obstruction can be performed via 2 approaches: an external nasal approach from the skin and an internal nasal approach from the nasal cavity. Both techniques have a history of over 100 years and are the gold standard approaches for the treatment of nasolacrimal duct obstructions. Alternatively, researchers have also attempted lacrimal passage reconstruction using various stents to restore the patency of the lacrimal passage, with nunchaku-type silicone tubes showing good surgical results according to several published reports. However, in cases in which the procedure required blind manipulation, the results were largely dependent on the surgeon’s skill. Under such circumstances, the dacryoendoscope was introduced at the beginning of this century and is currently widely used. In other fields, especially in gastroenterology, the introduction of gastrointestinal endoscopes that enable observation and treatment of lesions under direct observation has dramatically improved the treatment of gastrointestinal disorders. Considering that the dacryoendoscope has become standardized over the past 20 years since its introduction, this review summarizes the current status of lacrimal passage treatment using the dacryoendoscope.

## Introduction

Conventional lacrimal passage examination consists mainly of ocular surface observation (volume and properties of the tears), palpation (reflux, firmness, and tenderness of the lacrimal sac), lacrimal irrigation (patency, connection between the upper and lower lacrimal canaliculus, time until reflux, and properties of the reflux fluid), penetration and identification of the obstruction site through blind probing, and dacryocystography. Nasal endoscopes have been used to observe the nasal cavity, with computed tomography (CT) and magnetic resonance imaging (MRI) being performed if mass lesions were suspected. To observe the lumen of the lacrimal passage, a dacryoendoscope is necessary.

The dacryoendoscope, which is now widely used in Japan, was developed by Suzuki and colleagues in 2002 (Suzuki T, et al. Lacrimal Duct Fiberscope. The 106th Annual Meeting of the Japanese Ophthalmological Society, Sendai, 2002). Before the introduction of the dacryoendoscope in Japan, Cohen had been the first to observe the lacrimal passage using a small cylindrical endoscope in 1979 [1], and by 1991, Ashenhurst had succeeded in attaching a video camera to an endoscope and observing the lacrimal passage on a monitor [2]. In 1997, Emmerich reported a lacrimal duct reconstruction surgery using a rigid fiber dacryoendoscope, which was the prototype of the currently widely used dacryoendoscope [3].

**Fig. 1:**
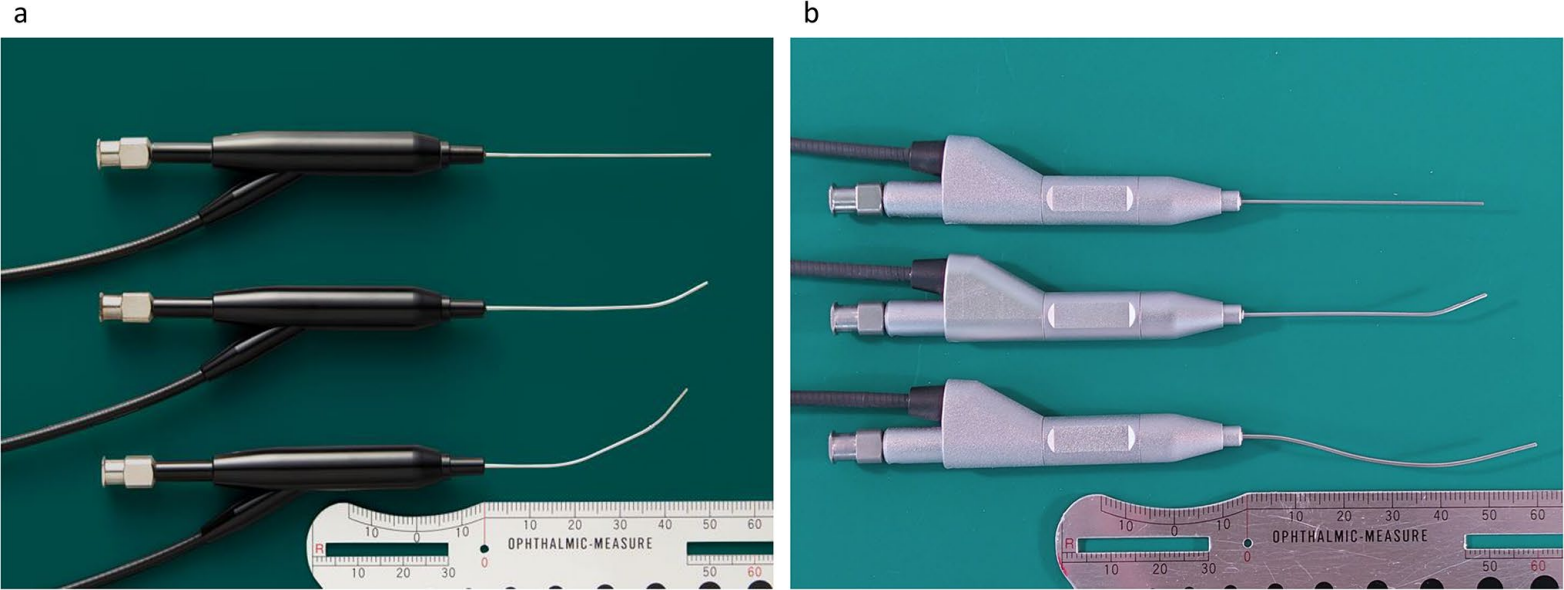
Dacryoendoscopes. **a** Straight type, bent-type, and double bent-type from above (Fibertec). **b** Straight type, bent-type, and double bent-type from above (Machida)

Nakamura and colleagues reported that on cone-beam CT dacryocystography, the line from the superior orbital rim through the common internal ostium (CIO) to the nasolacrimal duct opening inclined anteriorly toward the contralateral side of the unilateral primary acquired nasolacrimal duct obstruction (PANDO) in 92% of Japanese patients [4]. Currently, the dacryoendoscopes available in Japan are manufactured by Fibertec and by Machida, with the bent-type dacryoendoscope, which bends 27° upward at 10 mm from the tip, being extensively used. The straight-type dacryoendoscope is used for cases in which the nasolacrimal duct inclines posteriorly, whereas the double bent-type dacryoendoscope, which is bent in 2 stages, is used for cases having a large forward protrusion of the brow area, allowing observation of the entire lumen of the lacrimal passage (Fig. 1).

The first dacryoendoscope to be developed, in 2002, had a resolution of 6000 pixels, which improved to 10,000 pixels in 2012 while keeping the outer diameter at 0.9 mm, resulting in clearer images. However, for cases in which operability is a priority, a dacryoendoscope with an outer diameter of 0.7 mm at the tip and a resolution of 3000 pixels has been used since 2015, although the observation range and images obtained were unsatisfactory. In 2020, owing to improvements in the lens on the tip, endoscopic images had a dramatic increase in the depth of focus, with an observation distance of 1.5 to 7 mm.

However, although dacryoendoscopes are excellent for observing the lumen of the lacrimal passage, they are not suitable for understanding the relationship with the surrounding lacrimal passage. In some cases, therefore, imaging examinations, such as CT or MRI, should be used concurrently for diagnosis. In addition, the color tone of dacryoendoscopic images should be considered. When a black lesion is observed through a dacryoendoscope, the image is rendered gray instead of black due to the autoexposure mode. Hence, care should be exercised when observing malignant melanomas through a dacryoendoscope.

## Indications of dacryoendoscopy for lacrimal drainage disorders

**Fig. 2:**
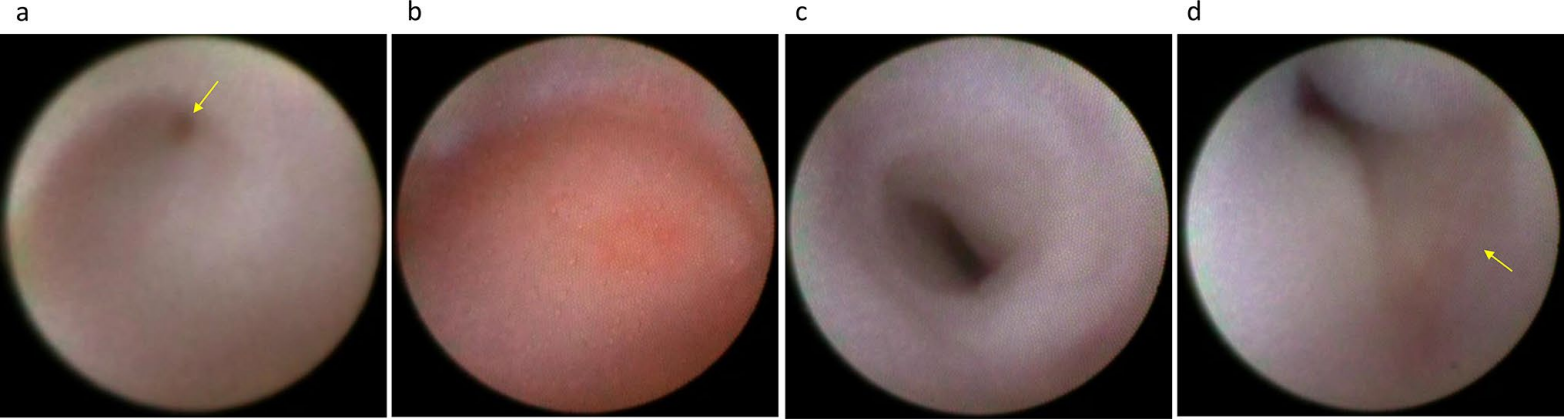
Endoscopic images of the canaliculus. **a** Common canalicular obstruction with dimple (yellow arrow). **b** Common canalicular obstruction without dimple. **c** Normal canaliculus. **d** Normal common canaliculus: lacrimal sac-like mucosa with blood vessels (yellow arrow) often observed at the common canaliculus adjacent to the CIO

**Fig. 3:**
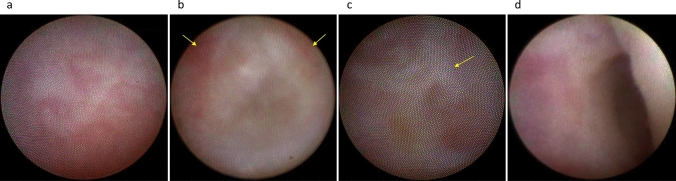
Endoscopic images of the lacrimal sac–nasolacrimal duct transition area. **a** Obstruction with abundant vessels. **b** Obstruction with easily hemorrhagic lesions (red patches) (yellow arrow). **c** Obstruction with fibrosis (white cord-like material) (yellow arrow). **d** Normal lacrimal sac–nasolacrimal duct transition

**Fig. 4:**
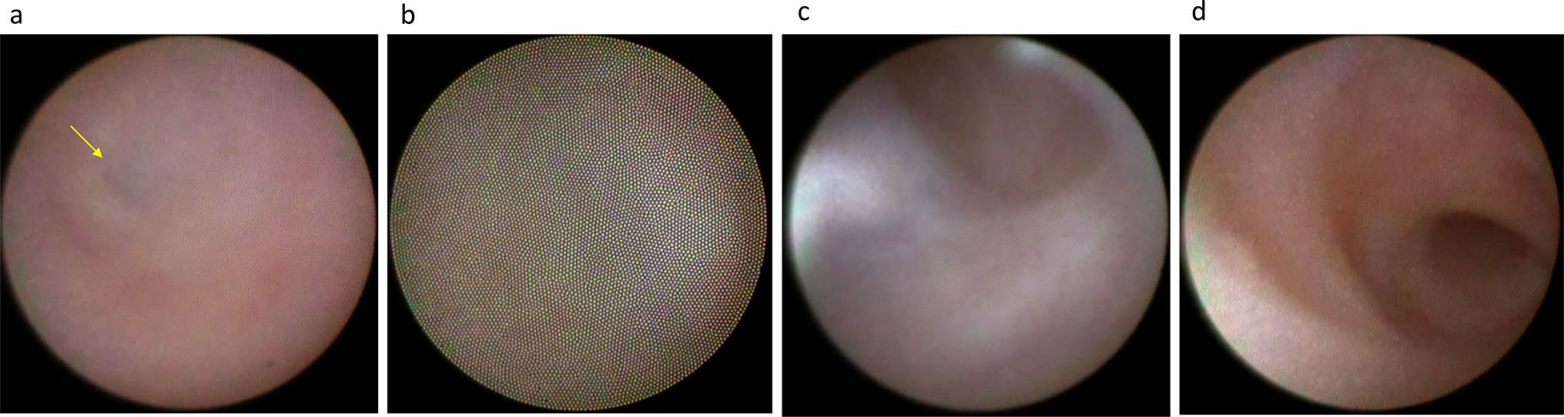
Endoscopic images of the nasolacrimal duct. **A** Obstruction at the middle portion of the nasolacrimal duct with dimples (yellow arrow). **b** Obstruction at the middle portion of the nasolacrimal duct without dimple. **c** Obstruction at the lower nasolacrimal duct. **d** Normal middle portion of the nasolacrimal duct

Although lacrimal irrigation can estimate the site of obstruction within the lacrimal passage, the accuracy of this approach is not necessarily high (around 70%) when compared with that of dacryoendoscopy [5, 6]. Another method for estimating the site of obstruction is dacryocystography; however, with this approach, the surgeon may sometimes struggle to determine whether the contrast agent has reached the site of obstruction [7]. Alternatively, dacryoendoscopic observation is superior in that it allows direct observation of the obstruction site, allowing detailed classification of not only the common canaliculus (Fig. 2a, b) or the lacrimal sac but also the nasolacrimal duct in terms of obstruction at the lacrimal sac–nasolacrimal duct transition (Fig. 3a–c) and the middle (Fig. 4a, b) or bottom (Fig. 4c) of the nasolacrimal duct. Furthermore, the degree of fibrosis in the obstruction (Fig. 3c) and inflammatory findings in the nasolacrimal duct mucosa (Fig. 3a, b) can be observed.

The diagnosis of a mass in the lacrimal passage is important. In a report of pathologic findings during dacryocystorhinostomy (DCR), 5.9% and 1.4% of patients presented with lesions (eg, granulation tissue or reactive lymphoid hyperplasia) and tumors, respectively, and 69% of the lesions diagnosed as tumors were malignant [8]. Some intralacrimal passage tumors cannot be identified using imaging tests, such as CT or MRI [9]. In such cases, observation of the lacrimal passage mucosa using a dacryoendoscope is expected for early detection of intralacrimal passage tumors. Although studies have used dacryoendoscopes to observe mass lesions, such as malignant melanomas [10], papillomas [11], and granulomas [12], further accumulation of knowledge is desired. The presence of a palpable mass at the inner canthus does not rule out the possibility of tumors outside the lacrimal passage, such as malignant lymphoma; thus, imaging studies, such as CT or MRI, are recommended [7, 13].

**Fig. 5:**
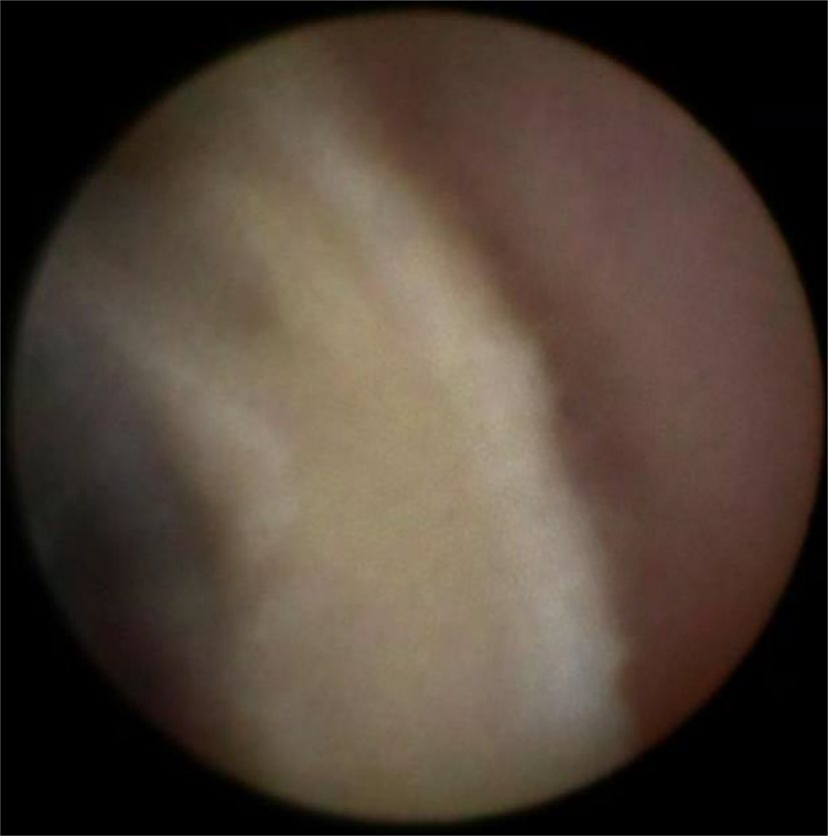
Dacryolithiasis. A yellowish dacryolith can be observed in the lacrimal sac

Besides tumors, foreign bodies in the lacrimal passage may be involved in epiphora and dacryocystitis. Most cases of canaliculitis are accompanied by canalicular concretions in which the most common pathogenic agents are *Actinomyces* species*;* thus, complete removal of the concretions is required for treatment. In such cases, dacryoendoscopy can be useful to confirm complete removal of the concretions [14]. Around 7.5% of cases with nasolacrimal duct obstruction develop dacryolithiasis, which can be a risk for acute dacryocystitis [15]. One study showed that among 23 cases of dacryolithiasis, twenty-one could undergo removal of dacryoliths through the nasal cavity via the nasolacrimal duct, whereas the remaining 2 cases required DCR owing to the large size of the dacryoliths [15]. Before surgery, dacryoendoscopy may facilitate efficient surgical method selection (Fig. 5). Studies have also reported on a punctal plug that had been inserted incorrectly into the canaliculus and was detected via dacryoendoscopy [7, 15]. An incorrectly inserted punctal plug may cause canaliculitis or dacryocystitis and should be removed through an incision made in the lacrimal punctum or discharged into the nasal cavity by use of a dacryoendoscope [15, 16].

## Operating the dacryoendoscope

In adults, dacryoendoscopic surgery can be performed under lacrimal passage mucosal anesthesia (4% lidocaine injected into the lacrimal passage). The use of a marked (or stopper) lacrimal dilator is recommended to dilate the lacrimal punctum as necessary. The incision should be made on the temporal side, not on the nasal side. The dacryoendoscope is then inserted after the lacrimal canaliculus has been straightened by pulling the eyelid laterally. The irrigation needs to be performed with a gradual flow of the irrigation fluid to avoiding a sudden increase in irrigation pressure, which may induce pain. The surgeon should avoid pushing the dacryoendoscope through the bending portion because the tip of the endoscope can easily damage the lacrimal passage mucosa. Given that the canaliculus mucosa is observed in white (Fig. 2c) and is susceptible to halation, the light intensity should be adjusted to lower than that used when the lacrimal sac is being observed.

Considering the possible difference in the angle between the bending portion at the lacrimal sac–nasolacrimal duct junction and the bending angle of the dacryoendoscope [17], surgeons should always consider the risk for lacrimal passage mucosal damage when advancing the endoscope forward at this point. If a risk for mucosal laceration formation exists owing to the difference in the angle, a Teflon sheath or similar device can be attached to the endoscope as appropriate to help it pass through the bending portion of the lacrimal passage [18]. Alternatively, several types of dacryoendoscopes with different angles could be prepared beforehand for a safer and smoother surgery.

## Lacrimal passage surgery using a dacryoendoscope

Lacrimal passage surgery using a dacryoendoscope can be performed under local anesthesia to the lacrimal passage mucosa, infratrochlear nerve anesthesia, infraorbital nerve anesthesia, and infiltration anesthesia surrounding the lacrimal passage. Infiltration anesthesia also involves the administration of a mucosal constrictor, such as epinephrine, and 4% lidocaine to the inferior meatus mucosa.

**Fig. 6:**
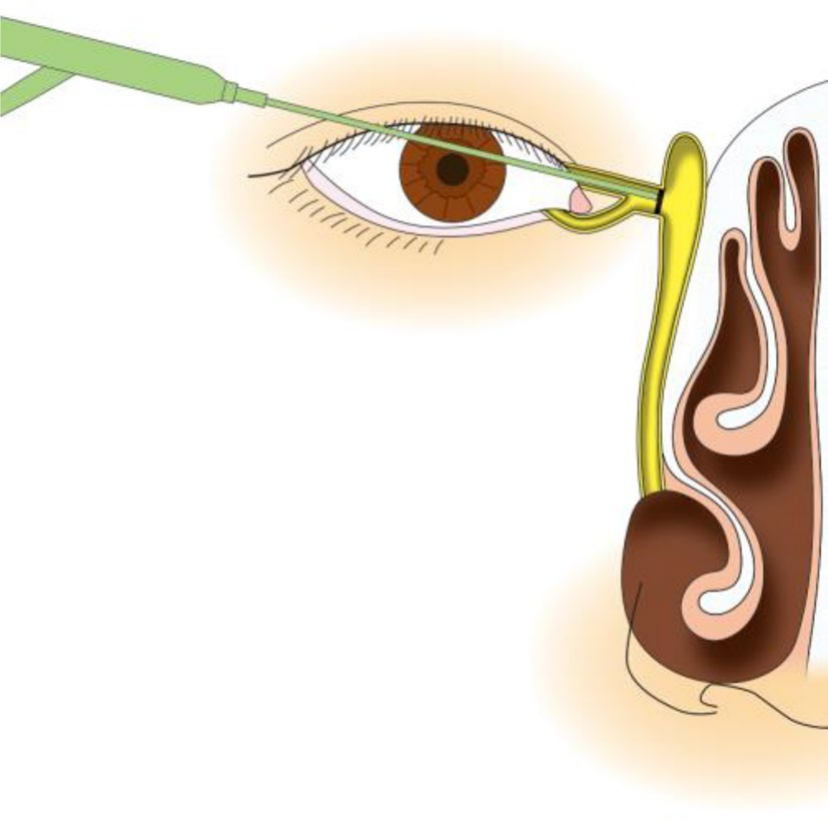
Schema showing direct endoscopic probing (DEP) of a common canalicular obstruction. The eyelid is pulled laterally, and the dacryoendoscope is pushed directly against the obstruction site to release it

**Fig. 7:**
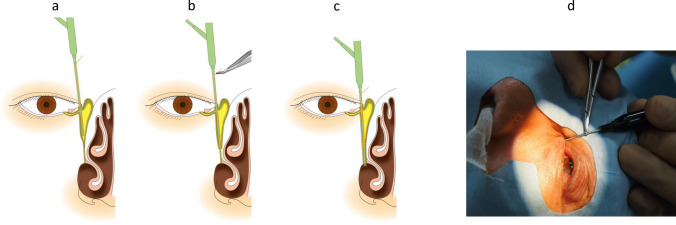
Schema and photograph showing sheath-guided endoscopic probing (SEP) of a nasolacrimal duct obstruction. **a** Observe the nasolacrimal duct obstruction. **b** Hold the brim of the Teflon sheath with forceps and keep the sheath 1–2 mm from the tip of the endoscope. **c** Keep the sheath protruding 1–2 mm from the tip of the endoscope and push the endoscope with the sheath together against the obstruction to release. **d** Photograph of the surgery

**Fig. 8:**
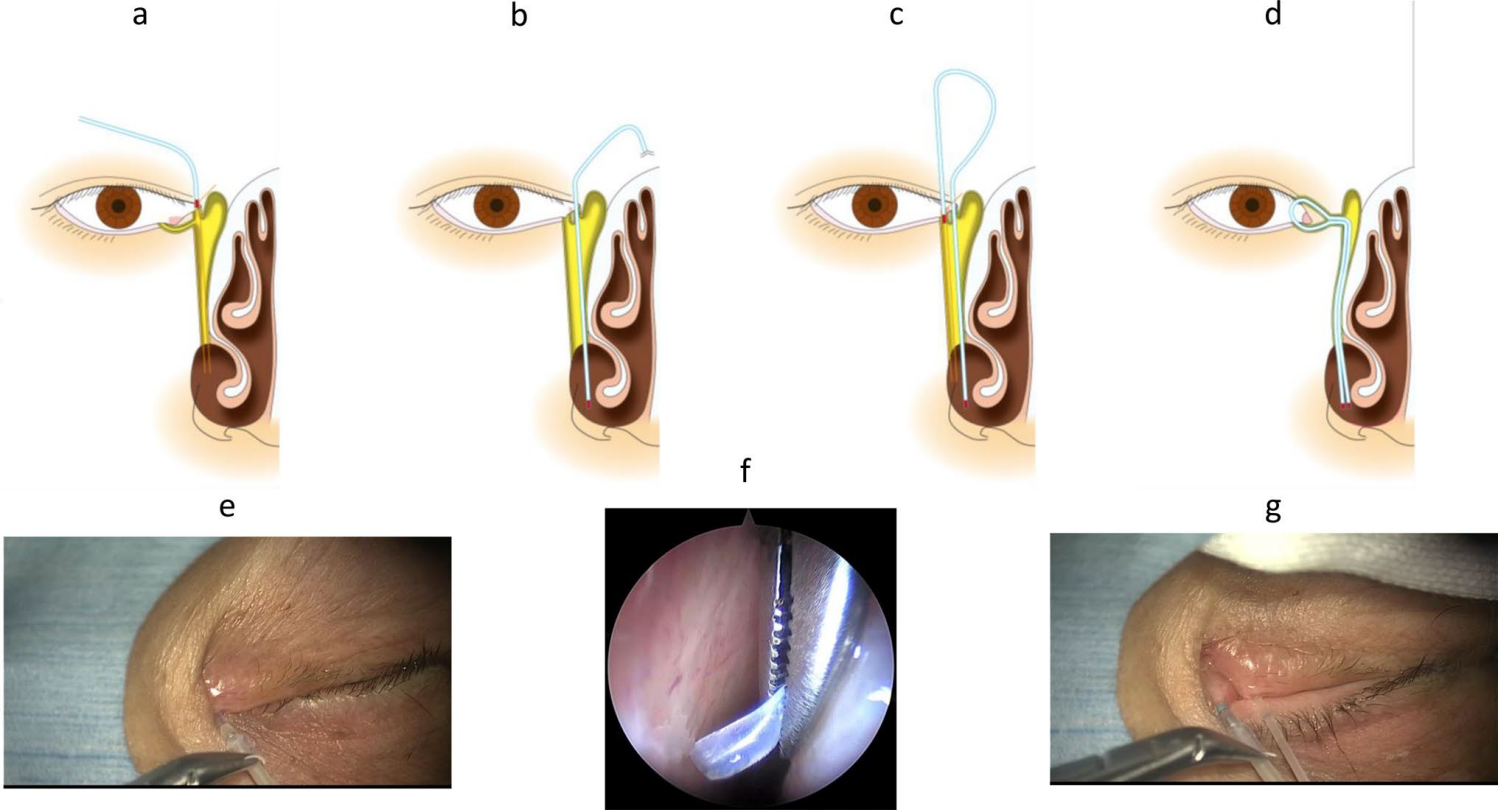
Schema and photograph showing sheath-guided intubation (SGI). **a** and **e** The stent tube is connected to the sheath on the punctum side, in which another side of the sheath has reached the inferior meatus. **b** and **f** The sheath is withdrawn from the inferior meatus by use of forceps, causing the tube to be drawn into the lacrimal passage. **c** and **g** The same procedure is performed on the other punctum. **d** Thereafter, the lacrimal passage obstruction is completely intubated

In the treatment of canaliculus and nasolacrimal duct obstructions using a dacryoendoscope in Japan, 2 methods for releasing the obstruction or stenosis can be used: direct endoscopic probing (DEP) [19] (Fig. 6) and sheath-guided endoscopic probing (SEP) [18] (Fig. 7). In DEP, the dacryoendoscope itself is used as a lacrimal probe. In SEP, a Teflon sheath is attached to the dacryoendoscope as an outer tube, after which the tip of the sheath is used to release the obstruction or stenosis. Nasolacrimal intubation is performed after the release of the obstruction or stenosis. Although direct nasolacrimal intubation has been used for the treatment of nasolacrimal obstruction, 1 study reported that 22% of cases experienced incorrect submucosal intubation [20]. To minimize the likelihood of submucosal incorrect intubation, sheath-guided intubation (SGI) was introduced [21]. In SGI, the sheath used for releasing the nasolacrimal obstruction is left in the lacrimal passage, the lacrimal tube is connected to the lacrimal sheath on the lacrimal punctal side, and the sheath is then drawn from the nasal cavity to guide the tube into the lacrimal passage (Fig. 8). Thus, SGI requires intranasal manipulation.

## Treatment for each portion of the lacrimal passage

### Treatment for lacrimal punctal obstruction

The difficulty in treating lacrimal punctal obstruction depends on the presence or absence of a lacrimal papilla and the etiology of the obstruction. Studies have reported that 86% of cases with congenital lacrimal punctal obstruction wherein the lacrimal papillae cannot be observed are accompanied by lacrimal canalicular hypoplasia [22]. Postepidemic keratoconjunctivitis (EKC), herpetic blepharoconjunctivitis, and anticancer drug-related lacrimal punctal obstruction have often been associated with canalicular obstruction, making restoration of the passage difficult. Cases with congenital or acquired lacrimal punctal obstruction in which lacrimal papillae can be observed, including other cases not mentioned above, can be treated by lacrimal punctal incision, lacrimal punctal dilation, and lacrimal intubation [23]. Outside Japan, the 3-snip procedure has been performed for the treatment of lacrimal punctal obstruction; however, because this technique causes significant changes in the structure of the vertical portion of the lacrimal canaliculus and punctum, the procedures mentioned above have been recommended for the treatment for lacrimal punctal obstruction [24]. Studies have also reported that in cases of recurrent lacrimal punctal obstruction, lacrimal intubation is recommended [25].

For details regarding the surgical procedures, a sharp-bladed knife is inserted vertically toward the eyelid at the center of the lacrimal papilla, followed by incision of the punctal mucosa. After the incision is made, the lacrimal punctum is gradually dilated by use of small to large lacrimal dilators. While the dilator is being advanced toward the horizontal portion of the lacrimal canaliculus, the eyelid should be pulled laterally to straighten the canaliculus. The presence of resistance at the tip of the dilator during dilation indicates a risk of damage to the lumen of the canaliculus. In such cases, observation of the canaliculus with the dacryoendoscope for the presence of other lesions in the lacrimal passage is recommended. Lacrimal intubation is recommended to prevent reocclusion. The tube is removed at 1 to 7 months after intubation [26–30], and treatment success rates of 81.8% to 100% have been reported at 3 to 12 months after removal of the tube [26–30].

### Treatment for lacrimal canalicular obstruction (grade 1)

Canalicular obstruction is usually classified on the basis of the Yabe–Suzuki classification: grade 1: a bougie can be inserted 11 mm or longer, and lacrimal irrigation shows communication between the upper and lower punctum (synonymous with common canalicular obstruction); grade 2: no passage between the upper and lower punctum, but a probe can be inserted 7 to 8 mm or longer; grade 3: obstruction more proximal than grade 2 [31].

**Fig. 9:**
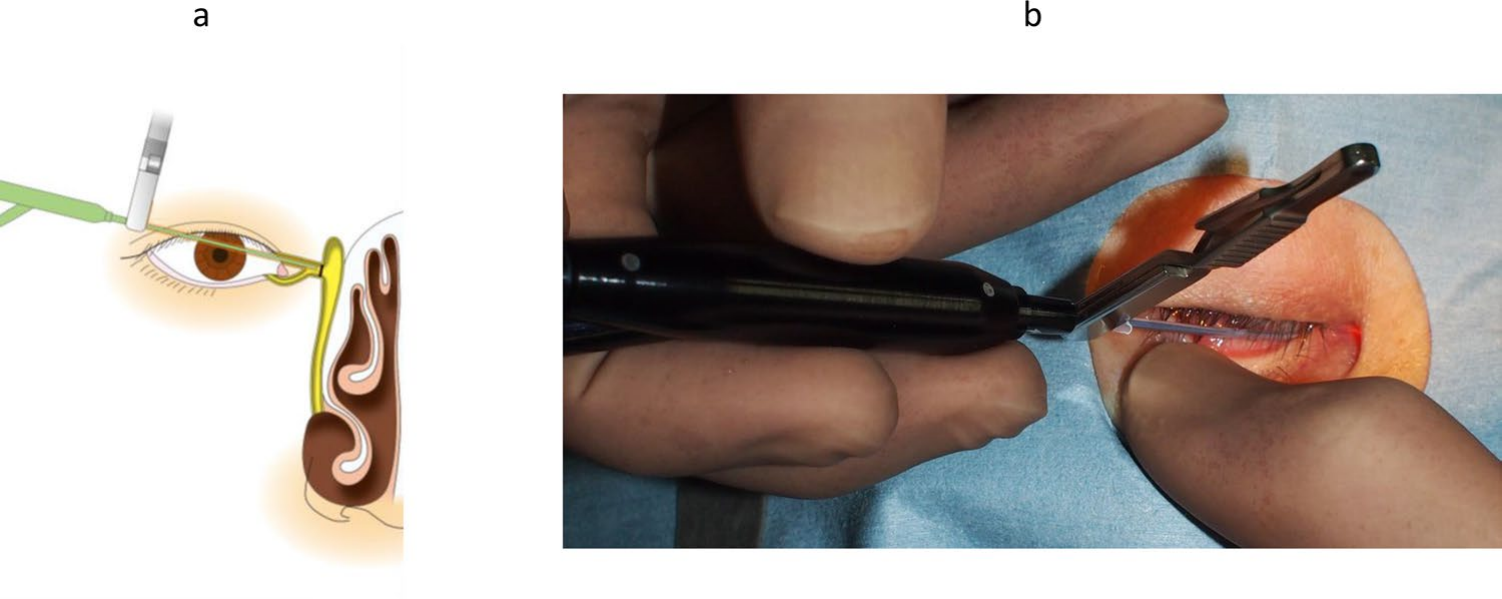
Schema and photograph showing sheath-guided endoscopic probing (SEP) of a common canalicular obstruction. **a** After confirming the obstruction of the common canaliculus, the sheath is protruded by around 3 mm from the tip of the endoscope and fixed by the lacrimal sheath stopper. **b** The eyelid is pulled laterally, and the endoscope is pushed against the obstruction to release it

For grade 1 canalicular obstructions, after punctal dilation, a dacryoendoscope is inserted to assess the obstruction site. While the eyelid is being pulled laterally, the obstruction is released using DEP (Fig. 6) or SEP. For SEP, the sheath is protruded 2 mm from the tip of the dacryoendoscope and fixed with the sheath stopper for easy manipulation [32] (Fig. 9). In DEP, after release of the obstruction site, the endoscope is pulled backward slightly to confirm the lacrimal sac lumen. After release of the obstruction, lacrimal intubation is performed using SGI. The tube is then removed at 2 to 10 months after intubation [26–30, 33–36].

Grade 1 canalicular obstructions are usually easy to restore owing to the short obstruction distance, although some cases present with an exceedingly difficult obstruction site that cannot be released through the use of SEP or DEP. In such cases, a metal lacrimal probe with a fine tip could be helpful.

One study using Kaplan–Meier analysis showed that grade 1 canalicular obstructions had a survival rate as good as 94% at a mean postoperative observation period of 878.3 days [37].

### Treatment for grade 2 and grade 3 lacrimal canalicular obstructions

Grade 2 or 3 canalicular obstructions according to the Yabe–Suzuki classification [31] are much more difficult to treat than are grade 1 obstructions. In particular, canalicular obstructions caused by anticancer drugs are often accompanied by lacrimal punctal obstruction or bilateral and simultaneous damage to the upper and lower canaliculi. According to Sakai and colleagues, approximately 60% of patients with anticancer drug-related lacrimal passage obstruction developed lacrimal punctal and canalicular obstructions [38]. Cases with a longer clinical course from the onset of lacrimal symptoms are more difficult to treat.

During the treatment of grade 2 or 3 canalicular obstructions using DEP or SEP, the presence of a small dent or pit after probing through the lacrimal sheath may indicate that the obstruction can be released. Even after releasing the obstruction, surgeons must confirm that the dacryoendoscope or sheath is inserted into the correct lumen because dacryoendoscopic images show white wall-like images owing to the long obstruction distance. The dacryoendoscope should be advanced gradually while the eyelid is strongly pulled laterally to keep the canaliculus straightened. A yellow color on the dacryoendoscopic images indicates submucosal orbital fat, and continued irrigation could potentially lead to eyelid edema. If releasing the obstruction is difficult or eyelid edema occurs, the surgery should be stopped at that point.

If either one of the upper or lower lacrimal canaliculus is obstructed or if only 1 side of the lacrimal canalicular obstruction can be released without release of the contralateral canaliculus, intubation with a unilateral lacrimal tube may be an option.

In cases with canalicular obstruction caused by anticancer drugs, 1 study recommended keeping the indwelling lacrimal tube during the periods of chemotherapy because removal of the tube during the chemotherapy is likely to cause reocclusion [38].

When both the upper and the lower canaliculi are obstructed and neither can be opened, various treatment strategies, including opening the lacrimal sac and probing through the CIO, conjunctivodacryocystorhinostomy (CDCR), conjunctivodacryocystostomy in which the upper portion of the lacrimal sac is anastomosed with the inferior conjunctival sac, have been reported [39–43].

### Treatment for nasolacrimal duct obstruction/stenosis

In nasolacrimal duct obstruction/stenosis, pus and mucus may accumulate in the lacrimal sac and nasolacrimal duct, and this should be washed out while a dacryoendoscope is being used to ensure visibility. Given that the lacrimal passage mucosa in patients with severe dacryocystitis may bleed easily, injecting lidocaine with epinephrine to stop the bleeding can help ensure visibility.

Nasolacrimal duct obstructions are released using DEP or SEP, and intubation is performed using SGI. As mentioned earlier, dacryolithiasis is a common complication of nasolacrimal duct obstruction. Accordingly, relatively small dacryoliths can be removed from the nasal cavity through the nasolacrimal duct by using the tip of the lacrimal sheath after release of the obstruction or stenosis followed by intubation with SGI. The tube is removed at 2 to 12 months after surgery [26–30, 35, 36, 44–47].

The surgical success rates of DEP or SEP and subsequent SGI is 70% to 87% at 1 year after tube removal [18, 19, 48]. The success rate of direct silicone intubation (DSI) for nasolacrimal duct obstruction is approximately 52.5% at 8 to 30 months after tube removal and 62.5% at 3 months [30], whereas DCR had a success rate of 90% to 99% [49–51]. Although lacrimal intubation via dacryoendoscopy has improved surgical outcomes when compared with that of DSI, its success rate is still not as high as that of DCR. Furthermore, the survival rate at 3000 days after tube removal is 64%, indicating a high risk of recurrence in the long-term postoperative period [46].

Only a few reports have shown good evidence regarding tube indwelling duration, highlighting the need for future randomized controlled trials (RCTs).

Factors associated with recurrence include a history of dacryocystitis [27, 28, 48], long disease duration [28, 47], long occlusion distances [29], and male sex [46–48].

### Treatment for congenital nasolacrimal duct obstruction

Congenital nasolacrimal duct obstruction (CNLDO) is a disease that resolves spontaneously at a higher rate [52]. However, when surgical treatment is required, probing should be considered the first choice [52]. Historically, probing had mainly been performed blindly. In recent years, however, several studies have successively reported on the usefulness of probing under visualization using a dacryoendoscope [53–60]. Consistent with this, the Japanese Society of Lacrimal Passage and Tear Dynamics published its clinical guidelines for CNLDO in 2024 [61]. In response to the clinical question regarding whether dacryoendoscopy is recommended for the treatment of CNLDO, the published guidelines have proposed the use of a dacryoendoscope for the probing of CNLDO. However, considering the high spontaneous resolution rate of CNLDO and the extremely limited number of facilities offering dacryoendoscopy in children, its use is proposed depending on the situation. According to the recommendation, probing by use of a dacryoendoscope is desirable, if possible. In particular, although additional blind probing among patients with an unsuccessful initial blind probing tended to have lower success rates [62–64], the use of a dacryoendoscope is expected to improve the success rate [53, 61]. An RCT investigating the usefulness of probing with a dacryoendoscope is currently ongoing under the initiative of the Japanese Society of Lacrimal Passage and Tear Dynamics.

CNLDO is sometimes accompanied by bony nasolacrimal duct obstruction or stenosis. Since a dacryoendoscope cannot release bony obstructions, it is not applicable in such cases. To confirm the absence of bony obstruction before surgery, preoperative head CT examination is recommended, especially in cases with facial deformity or a family history of refractory nasolacrimal duct obstruction [65].

Regarding endoscopic probing of CNLDO (6–74 months of age), although most studies have reported performing the procedure with the patient under general anesthesia [55–60], some have reported performing it with the patient under local anesthesia [53, 54]. On the basis of these studies, we can surmise that endoscopic probing of CNLDO is generally performed under general anesthesia but may be performed under local anesthesia provided that the child is old enough to control his/her body movements (generally 1 year and 3 months or younger) and the surgeon is skilled enough to perform it under a safe environment.

In Japan, a bent-type dacryoendoscope with a bent tip is commonly used, with reported success rates as good as 92.3–100% when probing for CNLDO [53–60]. Moreover, regarding safety, no serious complications have been reported thus far.

CNLDO generally refers to a membranous obstruction of the nasolacrimal duct opening into the nasal cavity [52, 55]. Therefore, the site of obstruction at the opening of the nasolacrimal duct should be identified and released, with care taken to avoid mucosal lacerations before the obstruction is reached. The opening of the nasolacrimal duct is often located near the medial wall of the lower end of the nasolacrimal duct [55]. Occasionally, dacryoliths can be observed, in which case the obstruction is released and then the dacryoliths are removed through the nasal cavity [55].

Lacrimal intubation after probing remains controversial. Previous studies on both probing alone [53, 56, 57, 59] and in combination with intubation [55, 58, 60] have been reported. In both treatments, treatment outcomes have been good, ranging from 92.3 to 100%, with the tubes being removed 4 to 8 weeks after the procedure [55, 58, 60]. Further studies are nonetheless needed to determine the appropriate tube indwelling time and whether the need for intubation differs depends on the condition of the obstruction, age in months, and/or type of surgery (initial or repeat).

### Treatment for acquired lacrimal passage obstruction in children

Regarding acquired lacrimal passage obstruction (ANDO) in children, 1 study reported that post-EKC secondary lacrimal passage obstruction is common in East Asia [66]. Unlike CNLDO, which is a membranous obstruction at the opening of the nasolacrimal duct into the nasal cavity, ANDO in children can occur at any site within the lacrimal passage and requires intubation or DCR. Moreover, treatment outcomes are poorer than those of CNLDO. One study reported that the treatment and management of ANDO in children are similar to those of PANDO in adults [67].

Despite the very few reports on the use of dacryoendoscope in the treatment of ANDO in children, leading to insufficient evidence, 1 study suggested avoiding DCR by identifying the site of obstruction through dacryoendoscopy and performing intubation properly [68]. The surgical technique is similar to that for adults but is more difficult owing to the narrower lacrimal passage and nasal cavity in children than in adults, as well as the frequent presence of canalicular obstruction.

## Complications

Lacrimal intubation with a dacryoendoscope is an excellent treatment method that can be performed with minimal invasiveness while observing the lumen of the lacrimal passage. This technique does not cause intraoperative or postoperative nasal bleeding, which can be problematic after DCR, or cerebrospinal fluid fistulas. However, to achieve safe and successful outcomes, various intraoperative and postoperative complications, which have been reported to include mucosal laceration, incorrect submucosal intubation, canalicular cheese wiring, and granulation [69], should be appropriately treated.

### Mucosal laceration and submucosal incorrect intubation

The most common complications encountered during intraoperative procedures using a dacryoendoscope and intubation are mucosal laceration and incorrect submucosal intubation. Understanding the anatomy of the lacrimal passage can help avoid these complications. In particular, poor visibility when passing through severe stenoses or long obstructions can cause mucosal lacerations and incorrect submucosal intubation. A yellow fatty mass in a bluish-white view on the dacryoendoscopic images is a very likely indicator that the tip of the dacryoendoscope has been inserted submucosally. These complications can be suspected if lacrimal passage bleeding or eyelid swelling due to irrigation is observed during dacryoendoscopy or intubation or if the patient complains of excessive pain [70]. Furthermore, in cases with a poor prognosis wherein epiphora and eye discharge continues after surgery, incorrect submucosal intubation should be suspected, for which another dacryoendoscopy is recommended [20].

Studies have reported that incorrect submucosal intubation tends to occur at the nasal-dorsal side of the angle formed between the lacrimal sac and the nasolacrimal duct. Careful follow-up is necessary because incorrect intubation can cause postoperative nasal bleeding [71]. Incorrect submucosal intubation can be easily identified as the formation of a mucosal bridge between the tubes on postoperative dacryoendoscopy [34].

### Canalicular cheese wiring: laceration of the lacrimal punctum

**Fig. 10:**
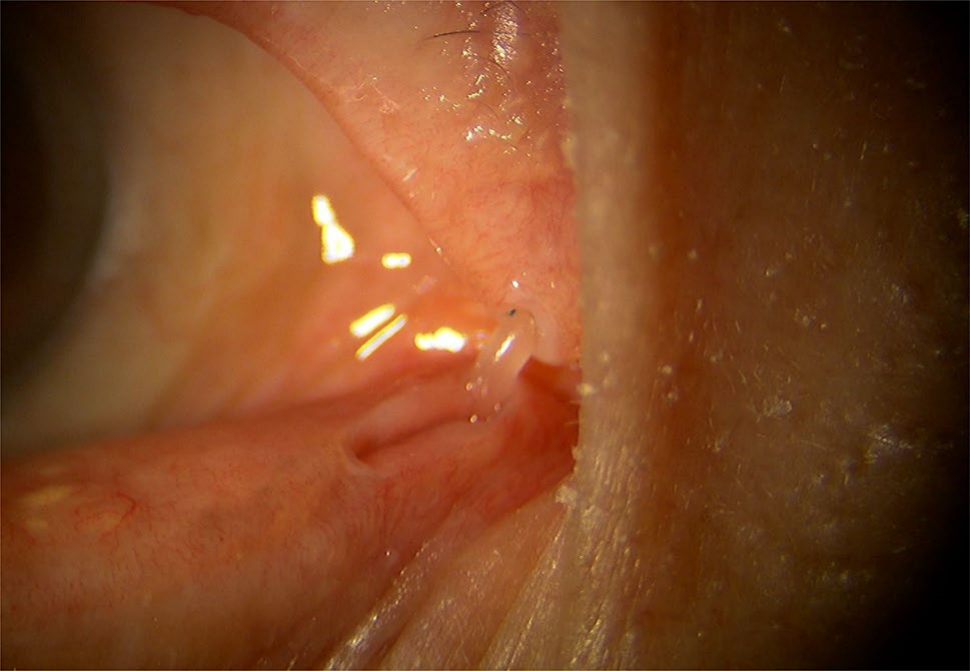
Canalicular cheese-wiring. Slit-like tear in the punctum enlarging in the direction of the horizontal part of the canaliculus

Canalicular cheese wiring is a phenomenon in which the lacrimal punctum tears in the direction of the canaliculus after intubation [69, 70, 72, 73] (Fig. 10), which can occur in both young and elderly patients [74–76]. The term *canalicular cheese wiring* was derived from the similarity of this condition to the use of a wire to separate Swiss cheese. Excessive lacrimal punctal incision or punctal dilation may cause the formation of canalicular cheese wiring [34, 71, 77]. Studies have also reported that incorrect submucosal intubation can prevent the smooth vertical movement of the tube during blinking owing to increased friction, increasing the risk for developing canalicular cheese wiring [69]. Another study reported canalicular cheese wiring complications in all 3 patients with prolonged (over 9 months) tube indwelling, highlighting the need to consider the tube indwelling time [74].

### Granulation tissue development in the lacrimal passage

A prolonged tube indwelling time may trigger the development of granulation tissues on the mucosa of the lacrimal passage owing to contact and friction with tubes, which may exacerbate epiphora and discharge. Granulation tissues can develop at any site within the lacrimal passage [20, 70, 75, 77, 78] and are often treated with corticosteroid eye drops. Dacryoendoscopy 1 month after tube removal often reveals the disappearance of the granulation tissues. This suggests that the development of granulation tissue may be a foreign body reaction to the tube.

### Dacryocystitis and corneal infection

A prolonged tube indwelling time may also cause bacterial growth and provoke infection in the lacrimal passage, resulting in chronic dacryocystitis [79] and occasionally infectious keratitis [80]. Reported culture results from corneal scrapings have detected *Moraxella lacunata*, *Streptococcus mitis*, *Neisseria cinerea*, *Pseudomonas aeruginosa*, α-hemolytic streptococci, *Pasteurella multocida*, and *Serratia bacteria* [35, 75, 76, 80].

Punctate superficial keratopathy and corneal erosions caused by friction with the tube and decreased tear fluid may cause infectious keratitis [81].

### Orbital cellulitis

Subcutaneous erythematous swelling with severe inflammation, mainly in the cheek area, may be observed within a few days of intubation. Studies have suggested that incorrect submucosal intubation causes orbital cellulitis [73, 82]. Intravenous and oral antibacterial treatment should be administered immediately, and if a tube is inserted, it should be removed immediately.

### Other problems related to intubation

Spontaneous removal of the tube or self-removal of the tube by the patient may often occur [76]. Loosening of the tube may occur postoperatively [71], which can cause a foreign body sensation and pain at the inner canthus, visual disturbances, conjunctival hyperemia, and corneal erosions. Therefore, the position of the tube should be corrected. If loosening of the tube recurs after correction, dacryoendoscopy or tube removal is recommended because it may have been caused by incorrect submucosal intubation.

In general, the tubes can be removed without resistance. Difficulties in the removal of the tube are often caused by incorrect submucosal intubation and, albeit less frequently, restenosis [73, 82]. Since the forceful removal of the tube may cause tearing of the tube, the tube ideally should be removed from the nasal cavity rather than forcibly removed from the lacrimal punctum. In general, a tube buried in the lacrimal passage should be removed via DCR.

## Summary

Over the past 20 years, the introduction of dacryoendoscopes that enable observation and treatment of lesions under direct vision has promoted a dramatic development in the treatment of lacrimal drainage disorders. In this review, we have summarized the usefulness and limitations of current treatment of lacrimal drainage disorders using dacryoendoscopes. Although the treatment of lacrimal drainage disorders using dacryoendoscopes has developed dramatically, some issues remain to be resolved, such as the tube indwelling duration and use of the dacryoendoscope for the probing of CNLDO. It is recommended that dacryoendoscopes can be used for the treatment of lacrimal drainage disorders understanding the benefits and limitations of such use.
